# Elevated cerebrospinal fluid levels of SERPIN E1 in participants with lewy body diseases

**DOI:** 10.1038/s41531-025-00984-3

**Published:** 2025-06-13

**Authors:** Milan Zimmermann, Madeleine Fandrich, Meike Jakobi, Benjamin Röben, Isabel Wurster, Stefanie Lerche, Claudia Schulte, Shahrzad Zimmermann, Christian Deuschle, Nicole Schneiderhan-Marra, Thomas O. Joos, Thomas Gasser, Kathrin Brockmann

**Affiliations:** 1 Department of Neurodegeneration and Hertie-Institute for Clinical Brain Research, Center of Neurology, University of Tuebingen, Tuebingen, Germany; 2 German Center for Neurodegenerative Diseases (DZNE), University of Tuebingen, Tuebingen, Germany; 3 NMI Natural and Medical Sciences Institute at the University of Tuebingen, Reutlingen, Germany

**Keywords:** Diagnostic markers, Diseases of the nervous system

## Abstract

Parkinson’s disease (PD) exhibits substantial phenotypic variability, likely influenced, at least in part, by proteins associated with pathways integral to aging processes. Plasminogen activator inhibitor-1 (SERPIN E1) is known for its association with aging processes and exacerbated α-Synuclein pathology. We examined whether SERPIN E1 levels in cerebrospinal fluid (CSF) differ among controls (CON, *N* = 16) and patients with PD (*N* = 479) or Dementia with Lewy bodies (DLB, *N* = 67), considering that these conditions represent a spectrum of α-Synuclein pathology. Kaplan-Meier survival analysis stratified by SERPIN E1 tertile levels was conducted to evaluate phenotype-modifying effects. Elevated levels of SERPIN E1 exhibited an association with increased age and lower MOCA scores. Heightened SERPIN E1 levels were observed in individuals diagnosed with DLB, followed by PD and CON, and in males compared to females. The quantification of SERPIN E1 in CSF could potentially serve as a surrogate marker, depicting (pathological) aging processes.

## Introduction

Parkinson’s disease (PD) serves as a prototype of age-related disorders, with its incidence increasing with age, thereby emphasising aging and its underlying pathways as a predominant risk factor for PD^1^. Furthermore, evidence from community-based longitudinal cohorts consistently indicates that both chronological age and an elevated age at disease onset independently predict cognitive decline, deterioration in motor function and increased mortality among individuals with PD ^2–4^. It is well-established that elements of blood coagulation, including plasminogen activator inhibitor-1 (SERPIN E1), demonstrate a notable age-related elevation. This phenomenon contributes to a procoagulatory state linked with advanced age^5^.

Growing evidence reveals other potential connections between blood coagulation and PD. Accumulation of α-Synuclein is a pathophysiological hallmark in PD, PD with Dementia (PDD) and Dementia with Lewy Bodies (DLB). Recently, a disease spectrum between PD, PDD and DLB based on α-Synuclein deposition was proposed, with the most pronounced Lewy body pathology in DLB patients^6^. This seems even more pronounced in patients carrying genetic variants in glucocerebrosidase 1 (*GBA1*)^7^. Within this framework, the nuanced platelet antiaggregant role of exogenous α-Synuclein, a protein also found in platelets, has been elucidated. This functionality primarily arises from the suppression of P-selectin expression on the platelet surface, thereby hindering thrombin-induced platelet activation^8^.

SERPIN E1 inhibits tissue-type plasminogen activator, thereby impeding the conversion of plasminogen to plasmin. Attenuated plasmin activity results in decreased fibrinolysis, consequently promoting the formation of blood clots. An additional important function of plasmin is to cleave α-Synuclein and impede its translocation from the extracellular environment to neighbouring cells. Both α-Synuclein and inflammatory processes have been demonstrated to upregulate the expression of SERPIN E1^9^, indicating a potential pathological feedback loop and promoting accumulation of α-Synuclein^10^.

Interestingly, possible pathophysiological links between SERPIN E1 and the other common aging-related neurodegenerative disorder, namely Alzheimer’s disease (AD), were found, too. Given the demonstrated contribution of concurrent AD pathology, coupled with additional vascular risk profiles, to cognitive decline in PD patients^11^, it is conceivable that these associations might also apply, at least in part, to PD and PDD. A function of plasmin is also to degrade Aß oligomers and monomers. Inhibition of SERPIN E1 let to reduced plasma and brain ß-amyloid levels, potentially ameliorating cognitive deficits in a mouse model of AD^12^. Furthermore, elevated levels of SERPIN E1 have been detected in plasma samples from PD patients^13^ and in CSF samples from AD patients^14^.

Here, we attempt to model this complex interplay between aging with SERPINE1 as proxy protein and neurodegeneration in a cohort of 479 patients with PD, 16 control participants (CON) and 67 patients with DLB.

Our aim is to explore whether CSF levels of SERPIN E1 are associated with progression of motor and/or cognitive impairment in PD. Patients were categorised into two groups: those with the *GBA1* variants (PD *GBA1*) and those with wildtype PD. This classification, coupled with the incorporation of a cohort of individuals diagnosed with DLB, was initiated to assess potential biological continuum patterns. This is motivated by the observation that patients with DLB exhibit elevated Aβ deposition in both the cortex and striatum, as well as an increased burden of cortical Lewy bodies in the temporal and parietal cortex when compared to PD^15^. In this line, we also considered common neurodegenerative proteins (ß-amyloid 1-42, h-TAU, p-TAU, NfL and α-synuclein), as they reflect different stages of neuronal damage and highlight additional Alzheimer’s pathology.

## Results

### Demographic and clinical characteristics

Of the 479 PD patients (PD total cohort), 313 were male (65.3%). Mean age at examination was 65.4 years (±9.9), mean age at onset 58.1 years (±10.5), mean disease duration 7.3 years (±5.2), mean MOCA score 25.3 (±3.9), mean UPDRS III score 26.1 (±11.5) and mean BDI II score 9.1 (±7.2) (Table 1).

**Table 1:** Demographic, clinical, and CSF biomarker data are presented as mean values with standard deviations (in parentheses) for patients with Parkinson´s disease (PD), Dementia with Lewy Bodies (DLB) and controls (CON)

		CON	PD			DLB		
			PD total	PD WT	PD *GBA1*	DLB total	DLB WT	DLB *GBA1*
Age at examination	**T**	65.6 (±9.5), *N* = 16	65.4 (±9.9), *N* = 479	65.9 (±9.9), *N* = 383	63.4 (±9.4), *N* = 96	72.5 (±6.5), *N* = 67^*^	73.6 (±6.4), *N* = 49^*^	69.4 (±6.0), *N* = 18
	**M**	68.4 (±7.8), *N* = 7	64.9 (±10.2), *N* = 314	65.6 (±10.2), *N* = 250	62.4 (±9.7), *N* = 64	70.9 (±5.6), *N* = 46	72.1 (±5.2), *N* = 30	68.7 (±5.8), *N* = 16
	**F**	63.4 (±10.5), *N* = 9	66.4 (±9.2), *N* = 166	66.7 (±9.4), *N* = 134	65.4 (±8.7), *N* = 32	76.0 (±7.1), *N* = 21^*^	76.0 (±7.3), *N* = 19^*^	75.3 (±5.3), *N* = 2
Age at onset	**T**		58.1 (±10.5), *N* = 479	58.8 (±10.5), *N* = 383	55.1 (±10.2), *N* = 96	69.3 (±7.3), *N* = 66	70.6 (±7.3), *N* = 48	66.1 (±6.2), *N* = 18
	**M**		57.8 (±10.7), *N* = 314	58.6 (±10.5), *N* = 250	54.5 (±11.1), *N* = 64	67.4 (±6.2), *N* = 45	68.6 (±6.1), *N* = 29	65.3 (±6.1), *N* = 16
	**F**		58.7 (±10.2), *N* = 166	59.3 (±10.6), *N* = 134	56.3 (±7.9), *N* = 32	73.5 (±7.7), *N* = 21	73.6 (±8.1), *N* = 19	72.5 (±3.5), *N* = 2
Disease duration	**T**		7.3 (±5.2), *N* = 479	7.1 (±5.0), *N* = 383	8.3 (±5.6), *N* = 96	3.2 (±2.1), *N* = 66	3.1 (±2.2), *N* = 48	3.4 (±1.8), *N* = 18
	**M**		7.2 (±4.9), *N* = 314	7.0 (±4.8), *N* = 250	7.9 (±5.2), *N* = 64	3.5 (±2.2), *N* = 45	3.6 (±2.5), *N* = 29	3.4 (±1.8), *N* = 16
	**F**		7.7 (±5.7), *N* = 166	7.4 (±5.5), *N* = 134	9.1 (±6.2), *N* = 32	2.4 (±1.5), *N* = 21	2.4 (±1.5), *N* = 19	2.8 (±1.7), *N* = 2
UPDRS III	**T**	1.3 (±2.6), *N* = 7	26.1 (±11.5), *N* = 446^§§^	26.0 (±11.5), *N* = 354^§§^	26.6 (±11.7), *N* = 92^§§^	26.8 (±9.2), *N* = 21^**^	25.5 (±7.9), *N* = 10^**^	28.0 (±10.4), *N* = 11^**^
	**M**	2.3 (±4.0), *N* = 3	26.7 (±11.5), *N* = 293^§^	26.9 (±11.5), *N* = 232^§^	25.9 (±11.5), *N* = 61^§§^	27.5 (±9.9), *N* = 17^*^	24.9 (±8.8), *N* = 8	29.8 (±10.8), *N* = 9
	**F**	0.5 (±1.0), *N* = 4	25.0 (±11.5), *N* = 154^§§^	24.3 (±11.2), *N* = 123^§§^	28.0 (±12.2), *N* = 31^§§^	24.0 (±5.0), *N* = 4	28.0 (±2.8), *N* = 2	20.0 (±1.4), *N* = 2
MOCA	**T**	27.7 (±2.2), *N* = 11	25.3 (±3.9), *N* = 403	25.4 (±3.6), *N* = 315	24.8 (±4.8) *N* = 88	15.1 (±4.3), *N* = 43^**^	14.6 (±4.0), *N* = 28^**^	16.0 (±4.7), *N* = 15^**^
	**M**	25.5 (±1.7), *N* = 4	25.3 (±3.6), *N* = 262	25.4 (±3.3), *N* = 203	24.8 (±4.5) *N* = 59	15.5 (±4.4), *N* = 31^**^	14.9 (±4.1), *N* = 17^**^	16.3 (±4.8), *N* = 14^**^
	**F**	29.0 (±1.3), *N* = 7	25.2 (±4.4), *N* = 142§	25.3 (±4.1), *N* = 113§	24.6 (±5.4) *N* = 29	13.9 (±3.8), *N* = 12^**^	14.1 (±4.0), *N* = 11^**^	12.0, *N* = 1
BDI II	**T**	4.5 (±6.2), *N* = 10	9.1 (±7.2), *N* = 348	8.9 (±7.1), *N* = 275	10.2 (±7.3), *N* = 73§	10.5 (±6.5), *N* = 6	12.0 (±6.0), *N* = 5	3.0, *N* = 1
	**M**	7.5 (±9.3), *N* = 4	9.0 (±6.7), *N* = 219	8.3 (±6.1), *N* = 170	11.6 (±7.9), *N* = 49	10.0 (±7.5), *N* = 3	10.0 (±7.5), *N* = 3	
	**F**	2.5 (±2.6), *N* = 6	9.3 (±7.9), *N* = 130	9.8 (±8.4), *N* = 106	7.4 (±5.0), *N* = 24	11.0 (±6.9), *N* = 3	15.0 (±0.0), *N* = 2	3.0, *N* = 1
ß-amyloid 1-42 (in pg/ml)	**T**	984.1 (±233.7), *N* = 14	719.9 (±269.4), *N* = 457^§§^	716.8 (±268.8), *N* = 364^§§^	732.2 (±272.9), *N* = 93^§§^	487.8 (±218.4), *N* = 66^**^	455.5 (±219.7), *N* = 48^**^	573.8 (±195.5), *N* = 18^**^
	**M**	948.7 (±292.7), *N* = 6	721.2 (±264.3), *N* = 300	717.0 (±262.7), *N* = 238	737.6 (±271.6), *N* = 62	479.2 (±203.9), *N* = 45^**^	419.7 (±187.9), *N* = 29^**^	587.1 (±191.8), *N* = 16
	**F**	1010.6 (±195.8), *N* = 8	718.8 (±279.5), *N* = 158^§^	718.1 (±280.5), *N* = 127^§^	721.3 (±279.7), *N* = 31^§^	506.1 (±251.0), *N* = 21^**^	510.2 (±256.6), *N* = 19^**^	468.0 (±268.7), *N* = 2
h-TAU (in pg/ml)	**T**	270.6 (±79.6), *N* = 14	249.7 (±130.5), *N* = 457	250.0 (±129.6), *N* = 364	248.8 (±134.7), *N* = 93	320.9 (±222.7), *N* = 66	356.7 (±242.0), *N* = 48	225.4 (±120.3), *N* = 18
	**M**	296.7 (±97.0), *N* = 6	236.2 (±117.9), *N* = 300	231.7 (±107.4), *N* = 238	253.5 (±151.4), *N* = 62	294.2 (±178.1), *N* = 45	335.4 (±191.0), *N* = 29	219.6 (±125.1), *N* = 16
	**F**	251.1 (±63.4), *N* = 8	276.5 (±148.8), *N* = 158	285.5 (±158.2), *N* = 127	239.3 (±94.3), *N* = 31	378.0 (±294.1), *N* = 21	389.2 (±307.1), *N* = 19	271.5 (±81.3), *N* = 2
p-TAU (in pg/ml)	**T**	48.9 (±14.8), *N* = 14	42.2 (±17.0), *N* = 446	42.6 (±17.0), *N* = 355	40.6 (±16.8), *N* = 91	46.0 (±25.8), *N* = 62	49.4 (±27.6), *N* = 45	36.9 (±17.8), *N* = 17
	**M**	51.3 (±20.7), *N* = 6	40.4 (±15.5), *N* = 294	40.3 (±14.6), *N* = 233	40.9 (±18.8), *N* = 61	43.6 (±25.3), *N* = 42	47.7 (±27.7), *N* = 27	36.3 (±18.8), *N* = 15
	**F**	47.0 (±9.5), *N* = 8	45.7 (±19.1), *N* = 153	47.0 (±20.3), *N* = 123	40.0 (±11.8), *N* = 30	51.1 (±26.8), *N* = 20	52.1 (±28.0), *N* = 18	41.5 (±9.2), *N* = 2
NfL (in pg/ml)	**T**	776.5 (±423.2), *N* = 10	969.6 (±797.6), *N* = 437	983.9 (±840.5), *N* = 347	914.6 (±605.6), *N* = 90	1709.9 (±1711.2), *N* = 60^*^	1843.5 (±1927.3), *N* = 43^*^	1372.0 (±937.0), *N* = 17
	**M**	1042.3 (±463.5), *N* = 5	976.9 (±618.8), *N* = 290	982.0 (±617.7), *N* = 231	957.1 (±628.2), *N* = 59	1636.6 (±1518.1), *N* = 40	1786.0 (±1767.0), *N* = 25	1387.6 (±980.5), *N* = 15
	**F**	510.6 (±107.1), *N* = 5	953.9 (±1064.9), *N* = 148	985.7 (±1162.3), *N* = 117	833.6 (±561.2), *N* = 31	1856.6 (±2080.3), *N* = 20^*^	1923.4 (±2181.0), *N* = 18^*^	1255.4 (±747.5), *N* = 2
α-Synuclein (in pg/ml)	**T**	652.0 (±237.9), *N* = 11	614.2 (±298.0), *N* = 451	630.9 (±306.3), *N* = 363	545.2 (±250.9), *N* = 88	515.5 (±302.0), *N* = 62	531.9 (±313.5), *N* = 45	471.9 (±273.2), *N* = 17
	**M**	583.2 (±181.7), *N* = 5	571.5 (±246.5), *N* = 296	579.1 (±250.5), *N* = 236	541.8 (±229.8), *N* = 60	472.9 (±283.9), *N* = 42	468.6 (±285.5), *N* = 27	480.8 (±290.8), *N* = 15
	**F**	709.4 (±279.5), *N* = 6	695.7 (±363.3), *N* = 156	727.0 (±370.1), *N* = 128	552.6 (±295.6), *N* = 28	604.8 (±326.3), *N* = 20	627.0 (±337.3), *N* = 18	405.4 (±19.1), *N* = 2
SERPIN E1 (in pg/ml)	**T**	687.6 (±326.1), *N* = 16	726.0 (±415.0), *N* = 479	737.8 (±429.8), *N* = 383	679.1 (±347.7), *N* = 96	986.6 (±747.5), *N* = 67	1074.7 (±840.4), *N* = 49	746.6 (±302.7), *N* = 18
	**M**	888.7 (±309.5), *N* = 7	771.9 (±453.6), *N* = 313	792.3 (±473.7), *N* = 249	692.7 (±357.0), *N* = 64	1021.1 (±778.9), *N* = 46	1165.7 (±908.8), *N* = 30	749.9 (±321.4), *N* = 16
	**F**	531.2 (±254.1), *N* = 9	639.5 (±313.6), *N* = 166	636.6 (±310.2), *N* = 134	651.8 (±332.1), *N* = 32	911.0 (±685.6), *N* = 21	931.1 (±719.3), *N* = 19	719.7 (±82.2), *N* = 2

Non-parametric Mann–Whitney-U test with significant *p* ≤ 0.007 (manual Bonferroni-correction) presented as following: § PD vs. CON; * DLB vs. CON; 0.001<*p* ≤ 0.007: §/*; *p* ≤ 0.001: §§/**.

*WT* wildtype, *GBA1* variant in the gene for glucocerebrosidase 1, *UPDRS III* Unified Parkinson’s Disease Rating Scale part III, *MOCA* Montreal Cognitive Assessment, *BDI II* Beck´s Depression Inventory II, *NfL* neurofilament light chain.

There were no relevant differences between male and female PD patients concerning mean age at examination (65.4 years (±9.9) vs. 64.9 (±10.2), *p* = 0.102).

Within the PD cohort, there were 59 individuals (12.3%) using acetylsalicylic acid, 4 individuals (0.8%) using a combination of acetylsalicylic acid and clopidogrel, 2 individuals (0.4%) using a combination of acetylsalicylic acid and ticagrelor, 1 individual (0.2%) using clopidogrel and 1 individual (0.2%) using rivaroxaban (a direct factor X inhibitor).

We observed a higher prevalence of blood-thinning medication intake, including inhibitors of platelet aggregation or anticoagulants, in PD patients with the highest levels of SERPIN E1 (lowest tertile: 10/139 (7.2%), mid tertile: 22/135 (16.3%), highest tertile: 35/138 (25.4%), *p* ≤ 0.001).

Higher CSF SERPIN E1 levels were associated with a higher age at examination (*r* = 0.464, *p* ≤ 0.001), a higher age at onset (*r* = 0.367, *p* ≤ 0.001), a longer disease duration (*r* = 0.155, *p* ≤ 0.001), a lower MOCA score (*r* = -0.249, *p* ≤ 0.001) and higher CSF levels of h-TAU (*r* = 0.353, *p* ≤ 0.001), p-TAU (*r* = 0.297, *p* ≤ 0.001), NfL (*r* = 0.404, *p* ≤ 0.001) and α-Synuclein (*r* = 0.250, *p* ≤ 0.001) (Table 2).

**Table 2:** Correlation analysis (Spearman correlation) between A

		A.SERPIN E1 CSF levels							B.age at examination						
		PD			DLB			CON	PD			DLB			CON
		total	WT	*GBA1*	total	WT	*GBA1*		total	WT	*GBA1*	total	WT	*GBA1*	
Age at examination	R	0.464	0.471	0.420	0.366	0.247	0.515	0.447							
	P	≤**0.001**	≤**0.001**	≤**0.001**	**0.002**	0.087	0.029	0.083							
	N	479	383	96	67	49	18	16							
Age at onset	R	0.367	0.376	0.323	0.335	0.225	0.465		0.884	0.893	0.858	0.964	0.943	0.948	
	P	**≤0.001**	**≤0.001**	**≤0.001**	**0.006**	0.124	0.052		**≤0.001**	**≤0.001**	**≤0.001**	**≤0.001**	**≤0.001**	**≤0.001**	
	N	479	383	96	66	48	18		479	383	96	66	48	18	
Disease duration	R	0.155	0.161	0.163	−0.014	0.014	0.117		0.141	0.143	0.150	−0.252	-0.292	−0.036	
	P	≤**0.001**	**0.002**	0.112	0.912	0.927	0.645		**0.002**	**0.005**	0.146	0.041	0.044	0.887	
	N	479	383	96	66	48	18		479	383	96	66	48	18	
MOCA	R	−0.249	−0.256	−0.198	−0.279	−0.295	−0.034	−0.600	−0.441	−0.460	−0.412	−0.407	−0.417	−0.274	−0.356
	P	**≤0.001**	**≤0.001**	0.064	0.070	0.127	0.904	0.051	**≤0.001**	**≤0.001**	**≤0.001**	**0.007**	0.027	0.323	0.282
	N	403	315	88	43	28	15	11	403	315	88	43	28	15	11
UPDRS III	R	0.081	0.128	−0.099	0.255	0.085	0.204	0.445	0.119	0.126	0.114	0.069	0.360	−0.213	0.401
	P	0.086	0.016	0.347	0.264	0.815	0.548	0.317	0.012	0.018	0.280	0.768	0.307	0.529	0.373
	N	446	354	92	21	10	11	7	446	354	92	21	10	11	7
BDI II	R	0.032	0.088	−0.113	0.464	0.051		0.343	−0.031	0.025	−0.229	0.203	0.359		0.257
	P	0.554	0.144	0.342	0.354	0.935		0.333	0.566	0.674	0.052	0.700	0.553		0.474
	N	348	275	73	6	5		10	348	275	73	6	5		10
ß-amyloid 1-42	R	0.077	0.074	0.126	0.050	0.059	0.278	0.253	−0.066	−0.036	−0.187	−0.119	0.008	−0.020	0.407
	P	0.098	0.160	0.229	0.692	0.690	0.265	0.383	0.160	0.497	0.073	0.340	0.958	0.938	0.149
	N	457	364	93	66	48	18	14	457	364	93	66	48	18	14
h-TAU	R	0.353	0.346	0.391	0.341	0.210	0.633	0.415	0.457	0.488	0.329	0.359	0.262	0.501	0.446
	P	**≤0.001**	**≤0.001**	**≤0.001**	**0.005**	0.153	**0.005**	0.140	**≤0.001**	**≤0.001**	**≤0.001**	**0.003**	0.072	0.034	0.110
	N	457	364	93	66	48	18	14	457	364	93	66	48	18	14
p-TAU	R	0.297	0.289	0.326	0.230	0.106	0.482	0.304	0.398	0.420	0.291	0.345	0.282	0.395	0.242
	P	**≤0.001**	**≤0.001**	**0.002**	0.072	0.487	0.050	0.291	**≤0.001**	**≤0.001**	**0.005**	**0.006**	0.061	0.117	0.404
	N	446	355	91	62	45	17	14	446	355	91	62	45	17	14
NfL	R	0.404	0.409	0.377	0.313	0.245	0.429	0.794	0.606	0.624	0.554	0.193	0.120	0.289	0.297
	P	**≤0.001**	**≤0.001**	**≤0.001**	0.015	0.113	0.086	**0.006**	**≤0.001**	**≤0.001**	**≤0.001**	0.140	0.445	0.260	0.405
	N	437	347	90	60	43	17	10	437	347	90	60	43	17	10
α-Synuclein	R	0.250	0.226	0.291	0.283	0.144	0.635	0.209	0.332	0.313	0.363	0.281	0.227	0.407	0.464
	P	**≤0.001**	**≤0.001**	**0.006**	0.026	0.345	**0.006**	0.537	**≤0.001**	**≤0.001**	**≤0.001**	0.027	0.133	0.105	0.151
	N	451	363	88	62	45	17	11	451	363	88	62	45	17	11

SERPIN E1 levels in cerebrospinal fluid (CSF) (in pg/ml) along with demographic data, clinical data and CSF biomarker levels (in pg/ml) in patients with Parkinson’s Disease (PD), Dementia with Lewy Bodies (DLB) and controls (CON). B. Age at examination is also analyzed in relation to the aforementioned parameters. Significant *p* ≤ 0.007 (manual Bonferroni-correction) are highlighted in bold. *R* correlation coefficient according to Pearson, *P* p-value, *N* sample size, *WT* wildtype, *GBA1* variant in the gene for glucocerebrosidase 1, *UPDRS III* Unified Parkinson’s Disease Rating Scale Part III, *MOCA* Montreal Cognitive Assessment, *BDI II* Beck’s Depression Inventory II, *NfL* neurofilament light chain.

Male PD patients demonstrated higher CSF levels of SERPIN E1 compared to female patients (771.9 (±453.6) vs. 639.5 (±313.6), *p* ≤ 0.001).

249 out of 383 (65.0%) PD WT patients were male. Mean age at examination was 65.9 years (±9.9), mean age at onset 58.8 years (±10.5), mean disease duration 7.1 years (±5.0), mean MOCA score 25.4 (±3.6), mean UPDRS III score 26.0 (±11.5) and mean BDI II score 8.9 (±7.1) (Table 1).

There was no significant difference between male versus female PD WT regarding the age at examination (65.5 years (±10.2) vs. 66.7 (±9.4), *p* = 0.268).

We found a higher prevalence of intake of blood-thinning medication in PD WT with the highest levels of SERPIN E1 (lowest tertile: 6/105 (5.7%), mid tertile: 18/110 (16.4%), highest tertile: 28/108 (25.9%), *p* ≤ 0.001).

Higher SERPIN E1 levels in CSF were associated with a higher age at examination (*r* = 0.471, *p* ≤ 0.001), a higher age at onset (*r* = 0.376 *p* ≤ 0.001), a longer disease duration (*r* = 0.161 *p* = 0.002), a lower MOCA score (*r* = -0.256, *p* ≤ 0.001) and higher CSF levels of h-TAU (*r* = 0.346, *p* ≤ 0.001), p-TAU (*r* = 0.289, *p* ≤ 0.001) and NfL (*r* = 0.226, *p* ≤ 0.001) (Table 2).

Male PD patients demonstrated significantly higher CSF levels of SERPIN E1 compared to female patients (792.3 (±473.7) vs. 636.6 (±310.2), *p* ≤ 0.001).

In PD *GBA1*, 64 out of 96 patients were male (66.7%). The mean age at examination was 63.4 years (±9.4), the mean age at onset 55.1 years (±10.2), the mean disease duration 8.3 years (±5.6), the mean MOCA score 24.8 (±4.8), the mean UPDRS III score 26.6 (±11.7) and the mean BDI II score 10.2 (±7.3) (Table 1**)**.

There was no significant difference between male versus female PD *GBA1* regarding the age at examination (62.4 (±9.7) vs. 65.4 (±8.7), *p* = 0.154).

There was no significant difference observed in terms of the use of blood-thinning medication (*p* = 0.435) between the SERPIN E1 tertile levels.

Higher CSF SERPIN E1 levels were associated with a higher age at examination (*r* = 0.420, *p* ≤ 0.001), a higher age at onset (*r* = 0.323, *p* ≤ 0.001) and higher CSF levels of h-TAU (*r* = 0.391, *p* ≤ 0.001), p-TAU (*r* = 0.326, *p* = 0.002), NfL (*r* = 0.377, *p* ≤ 0.001) and α-Synuclein (*r* = 0.291, *p* = 0.006) (Table 2).

We observed no statistically significant differences between male and female patients (*p* = 0.172), with respect to CSF protein levels of SERPIN E1. Furthermore, no relevant differences were identified in CSF SERPIN E1 levels based on the severity of *GBA1* variants (risk: 709.0 (±363.6), mild: 688.5 (±364.0), severe: 621.0 (±311.1), *p* = 0.952).

Out of 67 patients diagnosed with DLB, 46 were male, constituting a prevalence of 68.7%. The mean age at examination for this group was 72.5 years (±6.5), which is notably higher than the age at examination in CON (*p* ≤ 0.001) and PD (*p* ≤ 0.001). The mean age at onset was 69.3 years (±7.3), the mean disease duration 3.2 years (±2.1), the mean MOCA score 15.1 (±4.3), the mean UPDRS III score 26.8 (±9.2) and the mean BDI II score 10.5 (±6.5). 18 patients had a variant in *GBA1* (26.9%) (Table 1). 88.9% (16/18) of these patients were male.

In DLB total cohort, higher CSF levels of SERPIN E1 were associated with a higher age at examination (*r* = 0.366, *p* = 0.002), a higher age at onset (*r* = 0.335 *p* = 0.006) and higher h-TAU levels in CSF (*r* = 0.341, *p* = 0.005).

In DLB *GBA1*, higher SERPIN E1 levels in CSF were associated with higher h-TAU levels (*r* = 0.633, *p* = 0.005) and higher α-Synuclein levels (*r* = 0.635, *p* = 0.006) (Table 2).

No statistically significant results were observed when comparing male and female patients (*p* = 0.089). We found statistically significant higher CSF levels of SERPIN E1 in DLB vs. PD (total cohort: *p* = 0.019, Cohen’s *d* = −0.431; WT: *p* = 0.005, Cohen’s *d* = −0.505; *GBA1*: *p* = 0.148, Cohen’s *d* = −0.207) considering age at examination, disease duration and sex as covariates.

Of the 16 control participants (CON), 7 were male (43.8%). The mean age at examination was 65.6 years (±9.5), the mean MOCA score 27.7 (±2.2), the mean UPDRS III score 1.3 (±2.6) and the mean BDI II score 4.5 (±6.2) (Table 1).

There were no significant differences concerning the age at examination between PD total cohort vs. CON (*p* = 0.935), PD WT vs. CON (*p* = 0.794) or PD *GBA1* vs. CON (*p* = 0.549).

Higher SERPIN E1 levels in CSF were associated with higher levels of NfL in CSF (*r* = 0.794, *p* = 0.006), a higher age at examination in female CON (overall: *r* = 0.447, *p* = 0.083; male: *r* = –0.429, *p* = 0.337; female: *r* = 0.883, *p* = 0.002) (Table 2).

### Group comparison of SERPIN E1 levels between CON, PD and DLB

**Fig. 1:**
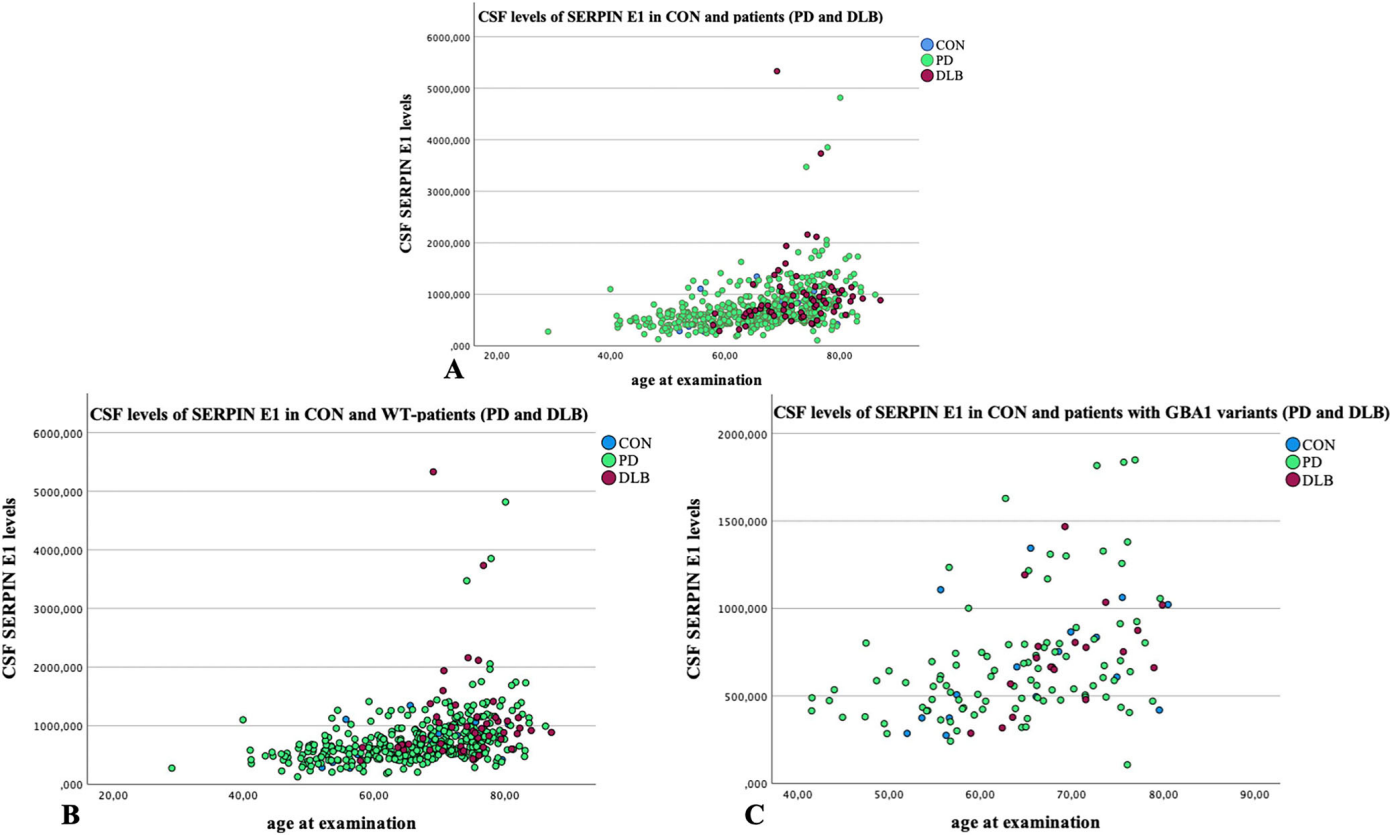
Levels of SERPIN E1 in CSF of patients and controls. Levels of SERPIN E1 in CSF are compared between controls (CON) (blue dots) and patients with Parkinson’s disease (PD) (green dots) and Dementia with Lewy Bodies (DLB) (red dots) (Fig. 2A), between CON and PD/DLB patients without GBA1 variants (wildtype, WT) (Fig. 2B), and between CON and PD/DLB patients with GBA1 variants (Fig. 2C). There was a significant difference between the three cohorts regarding CSF SERPIN E1 levels with the highest levels in DLB total cohort, followed by PD total cohort and CON.

There was a significant difference between the three cohorts regarding CSF SERPIN E1 levels with the highest levels in DLB total cohort (986.6 pg/ml (±747.5)), followed by PD total cohort (726.0 pg/ml (±415.0)) and CON (687.6 pg/ml (±326.1)) (p ≤ 0.001). Post-hoc tests revealed a significant difference in SERPIN E1 levels between DLB total cohort vs. PD total cohort (*p* ≤ 0.001) as well as between DLB WT vs. CON (*p* = 0.011) (Supplementary Table 1, Fig. 1.

### Longitudinal analysis

The incidence of cognitive impairment and postural instability did not exhibit statistically significant differences among the three CSF SERPIN E1 tertile groups throughout the longitudinal study in PD total cohort, PD WT and PD *GBA1*. The time interval until 50% of PD patients reached cognitive impairment did not show any statistically significant differences based on the SERPIN E1 tertiles. The follow-up time (in years) exhibited marginal statistically significant differences among the three tertiles of CSF SERPIN E1 levels in PD total cohort (lowest tertile: 9.0 (±4.8), *N* = 85, mid tertile: 9.4 (±6.3), *N* = 86, highest tertile: 9.2 (±5.7), *N* = 84, *p* = 0.029). Furthermore, patients with the highest SERPIN E1 tertile levels presented with a higher age at examination (Supplementary Tables 2 and 3).

## Discussion

Elevated levels of SERPIN E1 in CSF were associated with an advanced age at examination in individuals with PD and in control participants as well as with male sex in PD.

We found a significant difference between the three cohorts regarding CSF SERPIN E1 levels with the highest levels in DLB, lower levels in PD and the lowest levels in CON, albeit with the limitation of a small number of control participants and an older age at examination of DLB patients vs. the other two cohorts.

This finding might mirror the continuum of Lewy body pathology observed in patients with PD and DLB^6^, with the most pronounced Lewy body pathology in DLB patients, especially when exhibiting variants in *GBA1*^7^. Remarkably, CSF SERPIN E1 levels also exhibited statistically significant differences between DLB and PD taking into account age at examination, disease duration and sex as covariates in the ANCOVA.

No significant differences were detected between PD and CON, with both cohorts exhibiting a similar age at examination, nor between PD *GBA1* and PD WT.

Moreover, we identified an association between elevated SERPIN E1 levels and a lower MOCA score, along with increased CSF levels of h-TAU, p-TAU, NfL, and α-Synuclein in PD and partly in patients with DLB. As a limitation, the correlation analyses predominantly yielded r values in the low to moderate range of strength.

Given that a similar pattern was observed in the correlation analysis between the age at examination and the clinical and CSF biomarker data, it is reasonable to attribute these findings to age-related effects. Previous studies have also investigated age-dependent changes in CSF biomarkers, revealing an age-dependent increase in TAU^16^, NfL^17^ and α-Synuclein^18^.

Interestingly, a significantly higher prevalence of blood-thinning medication intake was observed in PD patients with the highest tertile of SERPIN E1 levels. This finding might be also attributed to the older age at examination and the subsequently increased prevalence of neurovascular diseases. Likewise, blood-thinning medication might directly influence SERPIN E1 levels.

It is pertinent to acknowledge that the identification of these age-related effects in our study serves not merely as a limitation but also as an opportunity. The quantification of these protein levels could potentially serve as surrogate marker, depicting (pathological) aging processes and helping to dissect different entities in the wide spectrum of Parkinson´s diseases. This aligns with the hypothesis of accelerated aging in PD, dementia, and other neurodegenerative conditions, especially in PD *GBA1*. Our findings may also facilitate the stratification of PD patients based on distinct risk scores for intervention studies.

As a limitation, no significant findings were observed in longitudinal analysis concerning the incidence of cognitive impairment and postural instability depending on CSF SERPIN E1 tertile levels. Additionally, there were no significant results regarding the time interval until 50% of PD patients developed cognitive impairment.

Another limitation of our current study is the use of both the UPDRS III and, since 2009, the MDS-UPDRS III, which has a broader scale.

The primary limitation of this study is the small number of control participants (*N* = 16), which restricts the ability to draw reliable conclusions about differences in protein levels. This limitation is particularly relevant when considering the potential utility of SERPIN E1 CSF levels as a diagnostic biomarker for early diagnosis. Given the scarcity of CSF samples from controls, future studies could focus on investigating potential differences in serum levels between controls and PD patients, which would also be more practical for use as a screening test.

The correlation between elevated SERPIN E1 levels and advanced age at examination, lower MOCA scores and higher levels of h-TAU, p-TAU and NfL, coupled with heightened SERPIN E1 levels in patients, may alternatively indicate potential risk characteristics concerning the course of PD associated with this protein. This interpretation aligns with the proposed pathophysiological role of SERPIN E1, which serves as an inhibitor of plasmin, thereby impeding the clearance of α-Synuclein.

The pathological feedback loop involving both α-Synuclein and neuroinflammation, leading to increased SERPIN E1 expression, could potentially exacerbate neurodegenerative processes, as previously suggested^10^. Given potential differences in age at examination between the cohorts, caution is warranted, necessitating validation in future studies. Nevertheless, our findings, which indicate the lowest levels of SERPIN E1 in CON, elevated levels in PD and the highest concentrations in DLB patients, may align with the current conceptualisation of a disease spectrum spanning PD, PDD and DLB.

In individuals with DLB, a heightened burden of Lewy bodies, particularly in the temporal and parietal cortex, is generally observed. Moreover, increased Aβ deposition in the cortex and striatum in DLB patients was noted post-mortem^15^. These findings may contribute to clinical distinctions such as an earlier onset of cognitive impairment and a higher frequency of visual hallucinations. In this context, the potential “synergistic” or “triggering” effects among cortical α-Synuclein pathology, p-TAU burden and Aβ deposition were explored^15^. The identification of a spectrum of SERPIN E1 levels across diseases with Lewy Body pathology may mirror the spectrum of heightened α-Synuclein and Aβ deposition due to pathological feedback loops and reduced clearance mediated by the inhibition of plasmin. Alternatively, it might mirror aging and thereby predisposes to these pathologies.

**Fig. 2:**
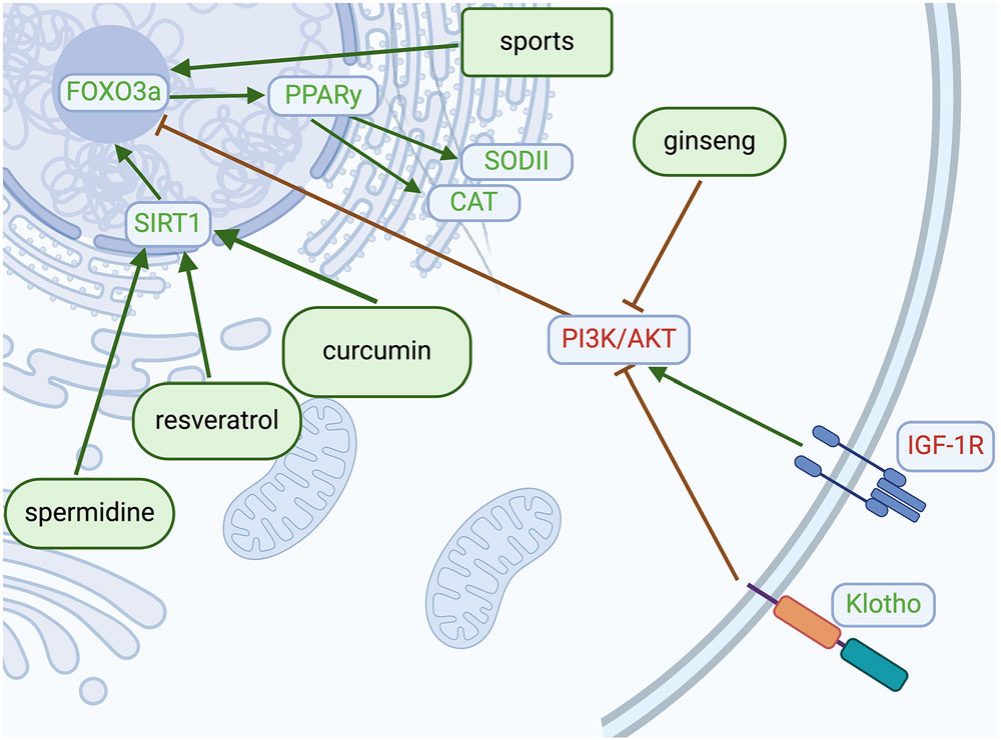
Klotho mitigates the phosphorylation of Forkhead box O3 (FOXO3a), primarily through the inhibition of Phosphoinositide 3-Kinase/Protein Kinase B (PI3K/AKT). This action prevents the displacement of this central transcription factor from the nucleus, resulting in elevated expression of antioxidative enzymes such as Superoxide Dismutase II (SODII) and human Catalase (CAT) through the activation of Peroxisome Proliferator-Activated Receptor γ (PPARγ). Possible outcomes include reduced production of interleukins, Major Histocompatibility Complex - II (MHC-II), Tumour Necrosis Factor α (TNF-α), and Reactive Oxygen Species (ROS), coupled with an increase in mitochondrial biogenesis and autophagy^25^. Similar effects can be induced by modifying lifestyle factors, including physical intervention, the application of substances such as ginseng, curcumin, or polyamines like spermidine, and caloric restriction, which has been associated with an extension in life expectancy^40^. These protective effects are hypothetically diminished by SERPIN E1 stabilising the Insulin-like Growth Factor 1 (IGF1) receptor and reducing telomere length^29^. Created with BioRender.com.

The adverse effects of SERPIN E1 on neurodegenerative diseases may also stem from its influence on one of the most relevant aging-related pathways encompassing the Insulin-like growth factor 1 (IGF1) - α-Klotho - SERPIN E1 - sirtuin 1 (SIRT1) - Forkhead box O3 (FOXO3a) - Peroxisome Proliferator-Activated Receptor γ (PPARγ) axis (Fig. 2). α-Klotho stimulates the transcription factor FOXO3a, resulting in an increased expression of antioxidative enzymes, such as superoxide dismutase II (SODII) and human catalase (CAT), facilitated by the activation of PPARγ, among other factors, through the inhibition of the phosphoinositide 3-kinase/protein kinase B (PI3K/AKT) pathway^19^. SIRT1 is also capable of enhancing the expression of PPARγ through the deacetylation of FOXO3a^20^.

α-Klotho-knockout mice manifest a phenotype indicative of premature aging, characterised by arteriosclerosis, osteoporosis, muscle atrophy, neuronal degeneration, a shortened lifespan and infertility ^21,22^. In PD research, both in vitro and in mouse models, the activation of PPARγ through the agonist pioglitazone has been demonstrated to result in a reduction of reactive oxygen species (ROS) ^23,24^. This reduction is accompanied by a decrease in inflammation^25^ and neuroinflammation, evidenced by lowered levels of interleukins, tumour necrosis factor α (TNF-α) and major histocompatibility complex - II (MHC-II), coupled with mitochondrial biogenesis and heightened autophagy ^26,27^.

Physiological, there is a transition from a predominant glucose metabolism in the growth hormone/IGF1 pathway to a prevailing lipid oxidation through the activation of PPARγ. This emerges as a pivotal pathway in longevity^25^. The positive impact on longevity and neurodegenerative diseases may stem from a potential overshoot in the heightened expression of antioxidative enzymes and increased autophagy surpassing the augmented ROS production during lipid metabolism as previously discussed for the protective effects of α-Klotho^28^.

In contradistinction to these protective effects, SERPIN E1 precipitates accelerated aging by reducing telomere length and stabilising the IGF1 receptor^29^.

Remarkably, discernible distinctions were noted between male and female patients in our current study. Male PD patients manifested higher SERPIN E1 levels than their female counterparts. This aligns with previous research demonstrating sex-specific variations in the central aging pathway. Such differences could be attributed to variations in insulin metabolism between sexes; for instance, FOXO1A exhibits high expression in the female reproductive system^30^. Moreover, sex-specific distinctions are observed in components of the coagulation cascade, potentially influenced by the effects of estrogen^31^, at least premenopausal.

We suggest future investigations in protein profiles of additional constituents within the central IGF1 – α-Klotho - SERPIN E1 - SIRT1 - FOXO3a - PPARγ - aging pathway, as well as the polyamine metabolism. Such explorations have the potential to furnish supplementary insights relevant to pharmacological interventions. Especially SIRT1^32^, PPARγ^33^ and SERPIN E1^34^ emerge as promising drug targets for patients with neurodegenerative and other age-related diseases. Importantly, protective effects on the central aging pathway, partly mediated by increased SIRT1 expression, can also be attained through lifestyle modifications, including physical interventions^35^, the use of ginseng^36^, curcumin ^37,38^, resveratrol^39^ or polyamines like spermidine^32^. Caloric restriction, which has shown an association with increased life expectancy^40^, is another noteworthy factor in promoting these protective effects.

To validate the results of our exploratory data analysis, additional studies focusing on these proteins within the central aging pathway, encompassing a larger cohort of control participants and patients with diverse neurodegenerative diseases, are imperative.

## Methods

### Participants and clinical investigations

All 479 PD patients (referred to as PD total cohort) and 67 patients with DLB were recruited and examined between 2001 and 2022 from the ward and outpatient clinic for PD at the University of Tuebingen. All patients were examined by a movement disorder specialist. The diagnosis of PD was defined according to UK Brain Bank criteria^41^. The following demographic and clinical data were obtained: age, sex, age at onset of parkinsonism. Additionally, we calculated the disease duration. We assessed the severity of motor symptoms using the Unified Parkinson´s Disease Rating scale part III (UPDRS III); from 2000 to 2008, and from 2009, the MDS-UPDRS were applied^42^. The clinical diagnosis of DLB was established in accordance with the revised consensus criteria for DLB, as outlined in the fourth report of the DLB consortium^43^. Cognitive function was tested using the Montreal Cognitive Assessment (MOCA)^44^ or the Mini Mental Status Examination (MMSE)^45^. As the MOCA has only been available since 2009, all MMSE scores were converted to MOCA equivalent scores according to an algorithm published recently^46^. MOCA cutoff ≤25 indicated cognitive impairment (point of maximum combined sensitivity and specificity^47^). Postural instability was characterised using the UPDRS III score, whereby patients demonstrating three or more compensatory steps in the pull test were classified as having postural instability. Depressive symptoms were assessed using Beck’s Depression Inventory (BDI) II^48^. The PD cohort, subjected to SERPIN E1 measurements, was chosen based on the criterion of possessing a comprehensive clinical data set. Of the 479 PD patients, 255 had longitudinal data pertaining to the onset of cognitive impairment, forming a smaller longitudinal sub-cohort.

All 16 control participants (CON) were spouses of patients with Parkinson´s disease or patients with functional disorders in whom neurodegenerative diseases have been extensively excluded on the ward.

All CSF samples were obtained from the Neuro-Biobank of the University of Tuebingen, Germany (https://www.hih-tuebingen.de/en/about-us/core-facilities/biobank/). The biobank is supported by the local University, the Hertie Institute and the DZNE.

### Collection of CSF Samples

Spinal tap was performed between 9:00 AM and 1:00 PM. Samples were directly taken from the bedside and centrifuged within 60 min after collection and frozen at −80 °C within 90 min after collection. Samples with abnormal routine CSF diagnostics (white blood cell count >6 cells/μL; Immunoglobulin subtype G index >0.7) were excluded.

### CSF measurements of ß-amyloid 1-42, h-TAU, p181-TAU (p-TAU), Neurofilament Light Protein (NfL) and α-Synuclein

CSF levels of ß-amyloid 1-42, h-TAU and p-TAU were measured using ELISA kits from INNOTEST, Fujirebio GmbH, Germany. CSF levels of NFL were measured using the UmanDiagnostics NF- light® assay. Intra-assay coefficients of variation for each CSF parameter were below 15%. CSF levels of total α-Synuclein were assessed using an ELISA kit for human α-synuclein (Roboscreen GmbH, Germany). Intra-assay imprecision of 4.4% was calculated from duplicate analyses and expressed as median of the range to average of the duplicates. Inter-assay imprecision of <10% was determined using two quality control CSF pool samples.

All measurements were performed by board-certified laboratory technicians who were blinded to the clinical data.

### Bead-based detection of SERPIN E1

SERPIN E1 CSF levels (in pg/ml) were detected using a commercial 3-plex human magnetic Luminex assay (R&D Systems, Wiesbaden, Germany, cat no. LXSAHM-03, lot number L139408). Individual analyses were performed with 1:2 diluted samples according to the manufacturer’s protocol. For the multiplex assay, 50 µL Luminex beads and 50 µL standards, quality control or CSF samples were pipetted into the wells of a 96-well plate. The specific analytes are bound by the immobilised antibodies on the colour-coded beads. Unbound sample was removed, and beads washed three times with wash buffer. 50 µL biotinylated detection antibody was added to each well and incubated at 800 rpm for 1 h at room temperature. The beads were washed three times to remove unbound detection antibody. 50 µL Streptavidin-PE conjugate was added to each well. After 30 min incubation at 800 rpm, unbound Streptavidin-PE was removed, and the beads washed three times. The beads were resuspended in 100 µl of buffer and then analyzed using the FlexMap 3D® analyser and Luminex xPONENT® 4.2 software (Luminex, Austin, TX, USA). Median fluorescence intensity (MFI) values were back-calculated using a 5PL regression fit to the standard samples (Bio-Plex Manager, version 6) to determine SERPIN E1 concentrations.

### Genetic analysis

DNA was isolated from blood collected in EDTA (ethylenediaminetetraacetic acid) tubes by salting out method and stored at 4 °C. Genetic screening for PD-associated mutations was done using the NeuroChip. The NeuroChip is a genotyping array (Illumnia) with a tagging variant backbone of ca. 306,670 variants. In addition, it contains 179,467 variants associated with neurological diseases^49^. Known pathogenic point mutations in the genes *GBA1*, *LRRK2*, *PRKN*, *PINK1* and *DJ1* detected by Neurochip were confirmed by Sanger sequencing. Multiplex ligation-dependent probe amplification was used to detect deletions and duplications in the genes *PRKN*, *PINK1* and *DJ1*.

We performed *GBA1*-subgroup classification in PD *GBA1* for mutation severity according to established mutational risk reported for PD: low risk (“risk”, *N* = 48), mild risk (“mild”, *N* = 20), severe risk (“severe”, *N* = 28). Of note, some variants that have been reported as nonrelevant for Gaucher disease have been proven to increase the risk for PD and therefore have been included in our analysis, for example, p. E326K and p.T369M.

### Statistics

Statistical analysis was performed using IBM-SPSS Statistics (IBM Corp. Released 2021. IBM SPSS Statistics for Macintosh, Version 28.0. Armonk, NY: IBM Corp.). Group comparisons were conducted among following groups: PD *GBA1* (*N* = 96), comprising patients with variants in *GBA1* gene, PD wildtype (WT, *N* = 383) (patients without variants in *GBA1*), PD total cohort (PD *GBA1* and PD WT, *N* = 479), CON (control participants, *N* = 16) and patients with Dementia with Lewy Bodies (DLB, *N* = 67).

The significance level was set at *p* ≤ 0.007 for cross-sectional analysis (manual Bonferroni-correction due to comparison of 7 groups) (Table 1 and Table 2), at*p* ≤ 0.016 for comparison of SERPIN E1 levels between CON, PD and DLB (Supplementary Table 1) and *p* ≤ 0.05 for longitudinal analysis (Supplementary Table 2).

Group comparisons for continuous variables of demographic, clinical and CSF data between DLB, PD and CON as well as between *GBA1* subgroups (cross-sectional analysis) were conducted using either the non-parametric Mann–Whitney U test or parametric analysis of covariance (ANCOVA), with covariates sex, age at examination and disease duration as appropriate.

Group comparisons of categorical variables (such as the incidence of cognitive impairment and postural instability; prevalence of intake of blood-thinning medication; prevalence of *GBA1* variants) were compared using the chi-squared test.

Spearman correlation analysis was employed to investigate the influence of SERPIN E1 levels and age at examination on demographic, clinical and biomarker data (Table 2).

Comparison of CSF SERPIN E1 levels between PD, DLB, and CON was performed using non-parametric Kruskal-Wallis test, followed by post-hoc tests (Supplementary Table 1). Additionally, SERPIN E1 levels between PD and DLB were also compared using ANCOVA with covariates sex, age at examination and disease duration. SERPIN E1 levels in controls were approximately normally distributed, as assessed by the Shapiro-Wilk test (*p* > 0.05), whereas this was not the case for PD or DLB patients (*p* ≤ 0.05).

Kaplan-Meier curves, along with Cox regression analysis incorporating the factor “tertile group”, were employed for longitudinal analysis. For these analyses, we compared the lowest vs. mid vs. highest tertile of CSF SERPIN E1 levels (Supplementary Table 2).

## Supplementary information

Supplemental Material

## Data Availability

The pseudonymized data of this study are available from the corresponding author upon reasonable request.

## Abbreviations

BDI II: Beck´s Depression Inventory II
CAT: human Catalase
CON: control participants
CSF: cerebrospinal fluid
DLB: Dementia with Lewy Bodies
FOXO3a: Forkhead box O3
GBA1: glucocerebrosidase 1
GH: Growth Hormone
IGF1: Insulin-like Growth Factor 1
MHC-II: Major Histocompatibility Complex - II
MMST: Mini Mental Status Test
MOCA: Montreal Cognitive Assessment
NfL: neurofilament light chain
PD: Parkinson´s Disease
PI3K/AKT: Phosphoinositide 3-Kinase/Protein Kinase B
PPARγ: Peroxisome Proliferator-Activated Receptor γ
SIRT1: sirtuin (silent mating type information regulation 2 homolog) 1
SODII: Superoxide Dismutase II
TNF-α: Tumor Necrosis Factor α
UPDRS III: MDS-UPDRS-part III; Movement-Disorder-Society-Unified Parkinson’s disease Rating Scale-part III
WT: wildtype

## References

[1] Bower, J. H., Maraganore, D. M., McDonnell, S. K. & Rocca, W. A. Incidence and distribution of parkinsonism in Olmsted County, Minnesota, 1976-1990. *Neurology* **52**, 1214–1220 (1999). 10214746 10.1212/wnl.52.6.1214

[2] Forsaa, E. B., Larsen, J. P., Wentzel-Larsen, T. & Alves, G. What predicts mortality in Parkinson disease?: a prospective population-based long-term study. *Neurology* **75**, 1270–1276 (2010). 20921512 10.1212/WNL.0b013e3181f61311

[3] Buter, T. C. et al. Dementia and survival in Parkinson disease: a 12-year population study. *Neurology* **70**, 1017–1022 (2008). 18362281 10.1212/01.wnl.0000306632.43729.24

[4] Alves, G., Wentzel-Larsen, T., Aarsland, D. & Larsen, J. P. Progression of motor impairment and disability in Parkinson disease: a population-based study. *Neurology* **65**, 1436–1441 (2005). 16275832 10.1212/01.wnl.0000183359.50822.f2

[5] Franchini, M. Hemostasis and aging. *Crit. Rev. Oncol. Hematol.* **60**, 144–151 (2006). 16860994 10.1016/j.critrevonc.2006.06.004

[6] Mensikova, K. et al. Lewy body disease or diseases with Lewy bodies?. *NPJ Parkinsons Dis.* **8**, 3 (2022). 35013341 10.1038/s41531-021-00273-9 PMC8748648

[7] Lerche, S. et al. Dementia with lewy bodies: GBA1 mutations are associated with cerebrospinal fluid alpha-synuclein profile. *Mov. Disord.* **34**, 1069–1073 (2019). 31189032 10.1002/mds.27731

[8] Acquasaliente, L. et al. Exogenous human alpha-Synuclein acts in vitro as a mild platelet antiaggregant inhibiting alpha-thrombin-induced platelet activation. *Sci. Rep.* **12**, 9880 (2022). 35701444 10.1038/s41598-022-12886-y PMC9198058

[9] Kim, K. S. et al. Proteolytic cleavage of extracellular alpha-synuclein by plasmin: implications for Parkinson disease. *J. Biol. Chem.* **287**, 24862–24872 (2012). 22619171 10.1074/jbc.M112.348128 PMC3408156

[10] Reuland, C. J. & Church, F. C. Synergy between plasminogen activator inhibitor-1, alpha-synuclein, and neuroinflammation in Parkinson’s disease. *Med. Hypotheses* **138**, 109602 (2020). 32035284 10.1016/j.mehy.2020.109602

[11] Smith, C. et al. Neuropathology of dementia in patients with Parkinson’s disease: a systematic review of autopsy studies. *J. Neurol. Neurosurg. Psychiatry* **90**, 1234–1243 (2019). 31444276 10.1136/jnnp-2019-321111

[12] Jacobsen, J. S. et al. Enhanced clearance of Abeta in brain by sustaining the plasmin proteolysis cascade. *Proc. Natl. Acad. Sci. USA* **105**, 8754–8759 (2008). 18559859 10.1073/pnas.0710823105 PMC2438386

[13] Pan, H. et al. Role of plasminogen activator inhibitor-1 in the diagnosis and prognosis of patients with Parkinson’s disease. *Exp. Ther. Med.* **15**, 5517–5522 (2018). 29844807 10.3892/etm.2018.6076 PMC5958833

[14] Sutton, R., Keohane, M. E., VanderBerg, S. R. & Gonias, S. L. Plasminogen activator inhibitor-1 in the cerebrospinal fluid as an index of neurological disease. *Blood Coagul. Fibrinolysis* **5**, 167–171 (1994). 8054448 10.1097/00001721-199404000-00002

[15] Jellinger, K. A. Dementia with Lewy bodies and Parkinson’s disease-dementia: current concepts and controversies. *J. Neural Transm* **125**, 615–650 (2018). 29222591 10.1007/s00702-017-1821-9

[16] Glodzik-Sobanska, L. et al. The effects of normal aging and ApoE genotype on the levels of CSF biomarkers for Alzheimer’s disease. *Neurobiol. Aging* **30**, 672–681 (2009). 17920160 10.1016/j.neurobiolaging.2007.08.019 PMC2774788

[17] Vermunt, L. et al. Age- and disease-specific reference values for neurofilament light presented in an online interactive support interface. *Ann. Clin. Transl. Neurol.* **9**, 1832–1837 (2022). 36196979 10.1002/acn3.51676 PMC9639622

[18] Winkel, I. et al. Cerebrospinal fluid alpha synuclein concentrations in patients with positive AD biomarkers and extrapyramidal symptoms. *J. Neural Transm.* **128**, 817–825 (2021). 34036433 10.1007/s00702-021-02351-x PMC8205875

[19] Lim, S. W. et al. Klotho enhances FoxO3-mediated manganese superoxide dismutase expression by negatively regulating PI3K/AKT pathway during tacrolimus-induced oxidative stress. *Cell Death Dis.* **8**, e2972 (2017). 28771227 10.1038/cddis.2017.365 PMC5596554

[20] Morselli, E. et al. Spermidine and resveratrol induce autophagy by distinct pathways converging on the acetylproteome. *J. Cell Biol.* **192**, 615–629 (2011). 21339330 10.1083/jcb.201008167 PMC3044119

[21] Kurosu, H. et al. Regulation of fibroblast growth factor-23 signaling by klotho. *J. Biol. Chem.* **281**, 6120–6123 (2006). 16436388 10.1074/jbc.C500457200 PMC2637204

[22] Kuro-o, M. et al. Mutation of the mouse klotho gene leads to a syndrome resembling ageing. *Nature* **390**, 45–51 (1997). 9363890 10.1038/36285

[23] Dias, V., Junn, E. & Mouradian, M. M. The role of oxidative stress in Parkinson’s disease. *J. Parkinsons Dis.* **3**, 461–491 (2013). 24252804 10.3233/JPD-130230 PMC4135313

[24] Richter, B., Haller, J., Haffner, D. & Leifheit-Nestler, M. Klotho modulates FGF23-mediated NO synthesis and oxidative stress in human coronary artery endothelial cells. *Pflug. Arch.* **468**, 1621–1635 (2016). 10.1007/s00424-016-1858-x 27448998

[25] van Heemst, D. Insulin, IGF-1 and longevity. *Aging Dis.* **1**, 147–157 (2010). 22396862 PMC3295030

[26] d’Angelo, M. et al. PPARγ and cognitive performance. *Int. J. Mol. Sci.* **20**, 5068 (2019). 31614739 10.3390/ijms20205068 PMC6834178

[27] Machado, M. M. F. et al. PPAR-gamma agonist pioglitazone reduces microglial proliferation and NF-kappaB activation in the substantia nigra in the 6-hydroxydopamine model of Parkinson’s disease. *Pharmacol. Rep.* **71**, 556–564 (2019). 31132685 10.1016/j.pharep.2018.11.005

[28] Zimmermann, M. et al. The longevity gene Klotho and its cerebrospinal fluid protein profiles as a modifier for Parkinson s disease. *Eur. J. Neurol.* **28**, 1557–1565 (2021). 33449400 10.1111/ene.14733

[29] Khan, S. S. et al. A null mutation in SERPINE1 protects against biological aging in humans. *Sci. Adv.* **3**, eaao1617 (2017). 29152572 10.1126/sciadv.aao1617 PMC5687852

[30] Li, Y. et al. Genetic association of FOXO1A and FOXO3A with longevity trait in Han Chinese populations. *Hum. Mol. Genet.* **18**, 4897–4904 (2009). 19793722 10.1093/hmg/ddp459 PMC2790334

[31] Abou-Ismail, M. Y., Citla Sridhar, D. & Nayak, L. Estrogen and thrombosis: a bench to bedside review. *Thromb. Res.* **192**, 40–51 (2020). 32450447 10.1016/j.thromres.2020.05.008 PMC7341440

[32] Jing, Y. H. et al. Spermidine ameliorates the neuronal aging by improving the mitochondrial function in vitro. *Exp. Gerontol.* **108**, 77–86 (2018). 29649571 10.1016/j.exger.2018.04.005

[33] Nowell, J., Blunt, E., Gupta, D. & Edison, P. Antidiabetic agents as a novel treatment for Alzheimer’s and Parkinson’s disease. *Ageing Res. Rev.* **89**, 101979 (2023). 37328112 10.1016/j.arr.2023.101979

[34] Kutz, S. M., Higgins, C. E. & Higgins, P. J. Novel combinatorial therapeutic targeting of PAI-1 (SERPINE1) gene expression in Alzheimer’s disease. *Mol. Med. Ther.* **1**, 106 (2012). 23847772 10.4172/2324-8769.1000106 PMC3703665

[35] Zeng, Z. et al. Exercise-Induced Autophagy Suppresses Sarcopenia Through Akt/mTOR and Akt/FoxO3a signal pathways and AMPK-Mediated mitochondrial quality control. *Front. Physiol.* **11**, 583478 (2020). 33224037 10.3389/fphys.2020.583478 PMC7667253

[36] Lim, S. W. et al. Ginseng increases Klotho expression by FoxO3-mediated manganese superoxide dismutase in a mouse model of tacrolimus-induced renal injury. *Aging* **11**, 5548–5569 (2019). 31400753 10.18632/aging.102137 PMC6710054

[37] Grabowska, W. et al. Curcumin elevates sirtuin level but does not postpone in vitro senescence of human cells building the vasculature. *Oncotarget* **7**, 19201–19213 (2016). 27034011 10.18632/oncotarget.8450 PMC4991376

[38] Khodavysi, M. et al. How can nanomicelle-curcumin modulate aluminum phosphide-induced neurotoxicity?: Role of SIRT1/FOXO3 signaling pathway. *AIMS Neurosci.* **10**, 56–74 (2023). 37077959 10.3934/Neuroscience.2023005 PMC10106336

[39] Stacchiotti, A. & Corsetti, G. Natural compounds and autophagy: allies against neurodegeneration. *Front. Cell Dev. Biol.* **8**, 555409 (2020). 33072744 10.3389/fcell.2020.555409 PMC7536349

[40] Bluher, M., Kahn, B. B. & Kahn, C. R. Extended longevity in mice lacking the insulin receptor in adipose tissue. *Science* **299**, 572–574 (2003). 12543978 10.1126/science.1078223

[41] Hughes, A. J., Daniel, S. E., Kilford, L. & Lees, A. J. Accuracy of clinical diagnosis of idiopathic Parkinson’s disease: a clinico-pathological study of 100 cases. *J. Neurol. Neurosurg. Psychiatry* **55**, 181–184 (1992). 1564476 10.1136/jnnp.55.3.181 PMC1014720

[42] Goetz, C. G. et al. Movement disorder society-sponsored revision of the unified Parkinson’s disease rating scale (MDS-UPDRS): scale presentation and clinimetric testing results. *Mov. Disord.* **23**, 2129–2170 (2008). 19025984 10.1002/mds.22340

[43] McKeith, I. G. et al. Diagnosis and management of dementia with Lewy bodies: Fourth consensus report of the DLB Consortium. *Neurology* **89**, 88–100 (2017). 28592453 10.1212/WNL.0000000000004058 PMC5496518

[44] Nasreddine, Z. S. et al. The montreal cognitive assessment, MoCA: a brief screening tool for mild cognitive impairment. *J. Am. Geriatr. Soc.* **53**, 695–699 (2005). 15817019 10.1111/j.1532-5415.2005.53221.x

[45] Folstein, M. F., Folstein, S. E. & McHugh, P. R. “Mini-mental state”. A practical method for grading the cognitive state of patients for the clinician. *J. Psychiatr. Res.* **12**, 189–198 (1975). 1202204 10.1016/0022-3956(75)90026-6

[46] Bergeron, D. et al. Multicenter validation of an MMSE-MoCA conversion table. *J. Am. Geriatr. Soc.* **65**, 1067–1072 (2017). 28205215 10.1111/jgs.14779

[47] Hoops, S. et al. Validity of the MoCA and MMSE in the detection of MCI and dementia in Parkinson disease. *Neurology* **73**, 1738–1745 (2009). 19933974 10.1212/WNL.0b013e3181c34b47 PMC2788810

[48] Beck, A. T., Steer, R. A. & Brown, G. K. *Manual for the Beck Depression Inventory-II*. Vol. (Psychological Corporation, 1996).

[49] Blauwendraat, C. et al. NeuroChip, an updated version of the NeuroX genotyping platform to rapidly screen for variants associated with neurological diseases. *Neurobiol. Aging* **57**, 247.e249–247.e213 (2017). 10.1016/j.neurobiolaging.2017.05.009 PMC5534378 28602509

