# Supplementary material for: Elevated cerebrospinal fluid levels of SERPIN E1 in participants with lewy body diseases
Source: NPJ Parkinsons Dis. 2025 Jun 13;11:166. doi: 10.1038/s41531-025-00984-3 (PMC12166053; doi:10.1038/s41531-025-00984-3)
Supplement: Supplementary file 1 — Supplemental Material [file 41531_2025_984_MOESM1_ESM.docx]

|  | Overall | | | | Male | | | | Female | | | |
| --- | --- | --- | --- | --- | --- | --- | --- | --- | --- | --- | --- | --- |
|  | DLB | PD | CON | p | DLB | PD | CON | p | DLB | PD | CON | p |
| total | 986.6*  (±747.5)  N=67 | 726.0 (±415.0)  N=479 | 687.6 (±326.1)  N=16 | ≤**0.001** | 1021.1* (±778.9)  N=46 | 771.9 (±453.6)  N=313 | 888.7 (±309.5)  N=7 | **0.004** | 911.0*§ (±685.6)  N=21 | 639.5 (±313.6)  N=166 | 531.2 (±254.1)  N=9 | **0.006** |
| WT | 1074.7*§  (±840.4)  N=49 | 737.8 (±429.8)  N=383 | 687.6 (±326.1)  N=16 | ≤**0.001** | 1165.7* (±908.8)  N=30 | 792.3  (±473.7)  N=249 | 888.7 (±309.5)  N=7 | **0.002** | 931.1*§ (±719.3)  N=19 | 636.6 (±310.2)  N=134 | 531.2 (±254.1)  N=9 | **0.009** |
| GBA1 | 746.6  (±302.7)  N=18 | 679.1 (±347.7)  N=96 | 687.6 (±326.1)  N=16 | 0.391 | 749.9 (±321.4)  N=16 | 692.7 (±357.0)  N=64 | 888.7 (±309.5)  N=7 | 0.110 | 719.7 (±82.2)  N=2 | 651.8 (±332.1)  N=32 | 531.2 (±254.1)  N=9 | 0.375 |

**Supplemental Table 1:** Comparison of CSF levels (in pg/ml) of SERPIN E1 between DLB (Dementia with Lewy Bodies), PD (Parkinson´s disease) and CON for total cohort, WT and *GBA1*. Kruskal-Wallis tests with significant p-values ≤0.05 indicated in bold letters. In case of a significant p-value, post-hoc tests were performed with a manual adapted significance level of p≤0.016 according to Bonferroni indicating significant results (PD vs. DLB: *; PD vs CON: &; DLB vs. CON: §).

|  | **CI overall** | **CI male** | **CI female** |
| --- | --- | --- | --- |
| **total cohort** | lowest tertile: 14.4 (11.8-17.0), (N=85)  mid tertile: 15.4 (11.2-19.6), (N=86)  highest tertile: 16.8 (13.6-20.1), (N=84)  p= 0.961 | lowest tertile: 12.8 (10.0-15.7), (N=29)  mid tertile: 13.0 (10.9-15.0), (N=68)  highest tertile: 14.5 (11.0-18.1), (N=64)  p= 0.674 | lowest tertile: 14.0 (11.3-16.6), (N=56)  mid tertile: 26.0 (12.9-39.0), (N=18)  highest tertile: 19.9 (15.2-24.6), (N=20)  p= 0.218 |
| **PD WT** | lowest tertile: 12.7 (10.3-15.1), (N=66)  mid tertile: 15.4 (10.7-20.1), (N=66)  highest tertile: 16.4 (13.4-19.3), (N=66)  p= 0.260 | lowest tertile: 9.9 (8.2-11.6), (N=21)  mid tertile: 12.8 (10.5-15.1), (N=51)  highest tertile: 13.9 (10.8-17.0), (N=51)  p= 0.264 | lowest tertile: 13.8 (10.7-16.8), (N=45)  mid tertile: 29.9 (18.8-40.9), (N=15)  highest tertile: 14.1 (11.3-16.9), (N=15)  p= 0.152 |
| **PD *GBA1*** | lowest tertile: 15.1 (11.8-18.3), (N=19)  mid tertile: 14.0 (10.1-17.8), (N=19)  highest tertile: 13.9 (8.2-19.7), (N=19)  p= 0.320 | lowest tertile: 15.7 (11.1-20.3), (N=9)  mid tertile: 14.3 (9.9-18.8), (N=16)  highest tertile: 12.7 (5.5-19.8), (N=13)  p=0.160 | lowest tertile: 14.5 (9.3-19.7), (N=10)  mid tertile: 12.9 (7.4-18.5), (N=3)  highest tertile: 15.9 (4.2-27.5), (N=6)  p= 0.726 |

**Supplemental Table 2:** Kaplan-Meier survival analysis and COX regression analysis for the time interval (in years) until 50% of the Parkinson´s disease (PD) patients reached the milestone cognitive impairment (CI) (MoCA≤25) in PD total cohort, PD wildtype (WT) and PD *GBA1*, stratified by tertiles of CSF SERPIN E1 levels. Categories: lowest tertile of SERPIN E1 CSF levels, mid tertile of SERPIN E1 levels, highest tertile of SERPIN E1 levels; 95% confidence intervals presented in brackets. P-values ≤0.05 are highlighted in bold. Abbreviations: *GBA1*: variant in the gene for glucocerebrosidase 1.

|  | **demographic and clinical data in the longitudinal sub-cohort** | | | | |
| --- | --- | --- | --- | --- | --- |
|  |  | lowest tertile | mid tertile | highest tertile | p value |
| **follow-up times** | PD total cohort | 9.0 (±4.8) | 9.4 (±6.3) | 9.2 (±5.7) | **0.029** |
|  | PD WT | 8.7 (±4.5) | 9.1 (±6.5) | 9.2 (±5.0) | 0.076 |
|  | PD *GBA1* | 10.0 (±5.4) | 10.0 (±6.0) | 9.6 (±7.7) | 0.939 |
| **age at examination** | PD total cohort | 58.2 (±9.2) | 62.0 (±9.1) | 66.4 (±8.5) | ≤**0.001** |
|  | PD WT | 58.6 (±8.6) | 62.2 (±9.2) | 66.8 (±8.8) | ≤**0.001** |
|  | PD *GBA1* | 57.4 (±10.9) | 59.3 (±10.0) | 65.8 (±6.2) | **0.012** |
| **incidence of**  **cognitive impairment** | PD total cohort | 33/85 (38.8%) | 38/86 (44.2%) | 34/84 (40.5%) | 0.766 |
|  | PD WT | 28/66 (42.4%) | 27/66 (40.9%) | 24/66 (36.4%) | 0.760 |
|  | PD *GBA1* | 6/19 (31.6%) | 10/19 (52.6%) | 10/19 (52.6%) | 0.323 |
| **incidence of**  **postural instability** | PD total cohort | 47/99 (47.5%) | 44/99 (44.4%) | 51/99 (51.5%) | 0.607 |
|  | PD WT | 34/77 (44.2%) | 32/76 (42.1%) | 36/76 (47.4%) | 0.805 |
|  | PD *GBA1* | 14/23 (60.9%) | 12/23 (52.2%) | 14/22 (63.6%) | 0.715 |

**Supplemental Table 3:** Demographic and clinical characteristics of Parkinson's disease (PD) patients stratified by PD total cohort, PD wildtype (WT) and PD *GBA1*, based on SERPIN E1 tertile levels in the longitudinal sub-cohort. Analysis of covariance (ANCOVA) incorporating age at examination, sex, and disease duration (follow-up times) or sex (age at examination) as covariates. Given the limited number of patients in PD *GBA1*, the non-parametric Mann-Whitney U test was conducted to compare follow-up times and age at examination in this sub-cohort. Chi-squared test employed for categorical variables, including the incidence of postural instability and cognitive impairment throughout the study duration. P-values ≤0.05 are highlighted in bold. Abbreviations: *GBA1*: variant in the gene for glucocerebrosidase 1.
